# Wisdom of the CROUD:  Development and validation of a patient-level prediction model for opioid use disorder using population-level claims data

**DOI:** 10.1371/journal.pone.0228632

**Published:** 2020-02-13

**Authors:** Jenna Marie Reps, M. Soledad Cepeda, Patrick B. Ryan

**Affiliations:** Janssen Research and Development Titusville, Titusville, NJ, United States of America; University of Queensland, AUSTRALIA

## Abstract

**Objective:**

Some patients who are given opioids for pain could develop opioid use disorder. If it was possible to identify patients who are at a higher risk of opioid use disorder, then clinicians could spend more time educating these patients about the risks. We develop and validate a model to predict a person's future risk of opioid use disorder at the point before being dispensed their first opioid.

**Methods:**

A cohort study patient-level prediction using four US claims databases with target populations ranging between 343,552 and 384,424 patients. The outcome was recorded diagnosis of opioid abuse, dependency or unspecified drug abuse as a proxy for opioid use disorder from 1 day until 365 days after the first opioid is dispensed. We trained a regularized logistic regression using candidate predictors consisting of demographics and any conditions, drugs, procedures or visits prior to the first opioid. We then selected the top predictors and created a simple 8 variable score model.

**Results:**

We estimated the percentage of new users of opioids with reported opioid use disorder within a year to range between 0.04%-0.26% across US claims data. We developed an 8 variable Calculator of Risk for Opioid Use Disorder (CROUD) score, derived from the prediction models to stratify patients into higher and lower risk groups. The 8 baseline variables were age 15–29, medical history of substance abuse, mood disorder, anxiety disorder, low back pain, renal impairment, painful neuropathy and recent ER visit. 1.8% of people were in the high risk group for opioid use disorder and had a score > = 23 with the model obtaining a sensitivity of 13%, specificity of 98% and PPV of 1.14% for predicting opioid use disorder.

**Conclusions:**

CROUD could be used by clinicians to obtain personalized risk scores. CROUD could be used to further educate those at higher risk and to personalize new opioid dispensing guidelines such as urine testing. Due to the high false positive rate, it should not be used for contraindication or to restrict utilization.

## Introduction

Opioid use disorder is defined as cognitive, behavioral and physiological symptoms indicating that the patient continues using the opioid despite significant opioid related problems. Opioid use disorder is a worldwide issue [1] estimated to have affected 2 million people in the USA in 2015 [2]. Opioid use disorder is a large contributing factor to opioid overdose and death in the USA [3]. Trends prior to 2014 show the overdose rate was increasing [4] although it seems to have stabilized more recently [5]. The ability to identify people at high risk of developing opioid use disorder before being prescribed their first opioid could be used to develop interventions, such as monitoring the high-risk subgroup, while ensuring those who require pain medication are not prevented access.

Numerous models have been developed that predict opioid use disorder in patients who are current opioid users [6–9]. Models incorporating predictors such as opioid prescribing patters in addition to demographics, non-opioid substance abuse, mental health conditions, hepatitis and cancer and have been able to predict future opioid use disorder with high discriminative ability (area under the receiver operating characteristic curve (AUC) as high as 0.93) [6,9]. However, a model that can predict opioid use disorder at the point a patient is first dispensed an opioid (i.e., when the patient is opioid naïve) has not been developed. A recent letter was published indicating the shortcomings of current opioid use disorder prediction tools and motivating the development of more advanced models [10].

In this paper we propose a novel perspective on the opioid use disorder prediction problem, where we use a cohort style design and start the prediction from the point the patient is first dispensed an opioid. This has two benefits; i) there is a clear point in time to apply our model (the visit when a clinician is about to dispense the first opioid to a patient) and ii) the candidate predictors will not include opioid use disorder traits, as the predictors are constructed using medical records up to the first opioid dispensing. We then follow the patients for 1-year to see whether each patient has opioid use disorder recorded. This design truly enables the development of a prognosis model to predict future opioid use disorder. We developed a score based prediction model named Calculator of Risk for Opioid Use Disorder (CROUD) that clinicians can use to predict a patient’s personalized risk of developing future opioid use disorder.

## Materials and methods

### OMOP common data model

As observational datasets are heterogeneous in content and format, code written to develop models on one dataset often cannot be shared to run on another dataset. To overcome the issue of format differences, the OMOP common data model (CDM) [11] was developed. The OMOP CDM provides a standardized format for observational healthcare data, requiring researchers to map their raw data into the OMOP CDM format, but once mapped, code can be directly shared with other researchers using different datasets. Studies have shown there is minimal data loss during the conversion [12] and the benefit from having a standardized format means network studies can be readily implemented [13]. For prediction, the standardized framework means it is easy to validate models across a diverse set of data.

### Data sources

All the data sources in this paper were mapped from their raw format into the OMOP CDM.

We used four US claims datasets for model development, see Table 1 for database details

**Table 1:** Database summary.

Data Source	Coverage	Data Type	No. of Patients	%		Time, year (y)	
				Female	Male	Start	End
Optum^©^ De-Identified Clinformatics^®^ Data Mart Database–Socio-Economic Status–(Optum)	USA–commercially insured and medicare	**Claims—**outpatient pharmacy, inpatient and outpatient medical claims, laboratory tests for a subset of the covered lives	93,423,000	54.0	56.0	2006	2018
IBM MarketScan^®^ Commercial Claims and Encounters Database (CCAE)	USA—commercially insured patients.	**Claims—**outpatient pharmacy, inpatient and outpatient medical claims, laboratory tests for a subset of the covered lives	142,660,000	51.2	48.8	2000	2018
IBM MarketScan^®^ Medicare Supplemental and Coordination of Benefits Database (MDCR)	USA—medicare supplemental coverage	**Claims—**outpatient pharmacy, inpatient and outpatient medical claims, laboratory tests for a subset of the covered lives	9,964,100	55.3	44.7	2000	2018
IBM MarketScan^®^ Multi-State Medicaid Database (MDCD)	USA–Medicaid patients	**Claims—**outpatient pharmacy, inpatient and outpatient medical claims	26,299,000	56.8	43.2	2006	2017

As each dataset contains diverse sets of patients, an opioid use disorder prediction model was trained on each dataset and validated on the other three datasets. We chose to develop a prediction model for each of the 4 datasets separately as this will have two potential consequences. Firstly, if one or more of the models transported well (has good consistent external AUC performance), then that model could be used and considered to be likely to be generalizable to a wide population. Alternatively, if none of the models transported consistently, then we could provide all models and use a person's health insurance type to determine which model is more suitable to apply for them.

The use of IBM MarketScan^®^ [14] and Optum [15] databases was reviewed by the New England Institutional Review Board (IRB) and were determined to be exempt from broad IRB approval. All data used in this study were fully anonymized before we accessed them.

### Prediction question

Our prediction question is within patients dispensed an opioid for the first time, predict who will have opioid use disorder recorded 1 day to 1-year from the first opioid dispensing.

We followed the published best practices for model development [16]. To enable full transparency and implementation by other researchers using different data all the definitions, analysis code and prediction models are provided in supplementary documents or in the OHDSI github repository.

### Target population: New users of opioids

Eligibility criteria to be in the target population is: A first-time opioid dispensing recorded with 3 years or more observation prior to the first opioid record, with no history of recorded opioid use disorder (abuse or dependence or unspecified drug abuse). By requiring 3 years or more observation prior we limit including people with a history of opioid use disorder who are new to the database. We also required that patients had a full 1-year post index follow-up or had opioid use disorder recorded within the year follow up. See S1 Appendix A for the codes and logic used to define the target population.

### Outcome

Opioid use disorder is defined as the recording of opioid abuse, dependency or treatment for opioid abuse in the claims data 1 day after first opioid dispensing until 365 days after first opioid dispensing. See S1 Appendix A for a list of codes used to define these terms.

### Candidate covariates

We derived ~80,000 candidate predictors from the administrative claims data that existed on or prior to the target index date. These variables were demographics, visit type, binary indicators of medical events and counts of record types. The demographics were gender, race, ethnicity, age in 5 year groups (0–4,5–9,10–14,…,95+) and month at the target index date. We created binary indicator variables for medical events based on the presence or absence of each concept within the OMOP CDM clinical domains of conditions, drugs, and procedures within two time periods: 365 days prior to index and all time prior to index. Both time prior covariates were considered in one single analysis. For example, there exists one covariate for each of ‘Diabetes mellitus’, ‘Hypertensive disorder’, and ‘Hypercholesterolemia’ (and all other diseases), based on the occurrence of a diagnosis code for each condition in the 365 days preceding the index date, and there also exists separate covariates for each of ‘Metformin’, ‘lisinopril’, and ‘HMG CoA reductase inhibitors’ (and all other drug ingredients and classes) based on the occurrence of a drug exposure record (outpatient pharmacy dispensing or inpatient procedural administration) for each drug in the 365 days prior to the index date.

As our binary covariates are the presence or absence of records for various conditions or drugs during time intervals prior to the opioid dispensing, we do not have missing values in the covariates. When a patient does not have a condition recorded, we cannot distinguish whether it is due to the patient having the condition but not having it recorded (missing) or them not experiencing the condition. Therefore, missing records for condition, drugs or procedures are treated as the patient not having the condition, drug or procedure. Age and gender are mandatory in the OMOP CDM, so are never missing. If a database contains race/ethnicity it will be recorded for all patients.

### Development and validation of prediction model

To develop the models, we sampled from the target population to create development data consisting of the predictors and labels indicating whether a patient had opioid use disorder recorded within a year. The development data were split into training data (75%) and testing data (25%). When externally validating the developed models we used the complete target population (we did not sample).

A logistic regression with LASSO regularization (LASSO logistic regression)[17] was trained, within each database, using 3-fold cross validation on the training data to find the optimal regularization value. 3-fold cross validation was chosen due to the large data size. A LASSO logistic regression is a slightly modified logistic regression model, where an additional cost term is added to the objective function corresponding to the model complexity (number of covariates in the model) and this causes the majority of covariates to drop out of the model. LASSO logistic regression is a good classifier to use when there is a large number of covariates [18]. We chose a LASSO logistic regression model rather than more complex machine learning models as we required a parsimonious model that could be readily used within a healthcare setting.

The testing data were used to internally validate the model. To evaluate the model's discrimination the area under the receiver operating characteristic curve (AUC) was used. The AUC value ranges from 0.5 (random guessing) to 1 (perfect discrimination) and is a measure of how well the prediction models ranks those with the outcome ahead of those without the outcome. The model's calibration was assessed by manually inspecting the calibration plot corresponding to the mean observed risk vs the mean predicted for ten predicted risk quantiles. If the model is well calibrated, then the mean observed risk should be approximately the same as the mean predicted risk for each quantile. We also externally validated each model by applying the model developed on one dataset to the other three datasets and calculated these measures.

In addition, we also investigated various sensitivity (percentage of people with opioid use disorder we identify with the model), specificity and positive predictive value (percentage of people the model predicts to have opioid use disorder who have opioid use disorder recorded) cut points to determine the clinical usefulness of the model.

### Calculator of Risk for Opioid Use Disorder (CROUD)

Machine learning models for predicting opioid use disorder are often complex and contain a large number of covariates. We sought to develop a simple model that could be used clinically for risk stratification (e.g., that could also identify subjects with different risks of developing opioid use disorder). To accomplish this we inspected the full model and selected a small number of variables that appear to be informative in terms of predicting opioid use disorder (the variables that had a high absolute coefficient, were selected across multiple models developed on the different databases and were recorded for a large number of patients). We developed CROUD by training a logistic regression model on Optum using the small number of variables and scaling the coefficients into integer points (we multiple each coefficient by a sufficiently large constant value and then rounded to get integer points). For example, if variable 1 had a coefficient 0.22, variable 2 had a coefficient 0.10 and variable 3 had a coefficient 0.04, then we could multiply the coefficients by 50 so the coefficients become 11, 5 and 2 respectively.

#### Sensitivity analysis

To evaluate how well the developed model performs on different types of patients we performed a sensitivity analysis across various target populations. We validated the model on patients who initiate opioids and become longer term users (those who have an opioid for 90 days or more) as these patients are probably more likely to develop opioid use disorder. We also validated the model on patients with less prior observation (only requiring 1 year of continuous enrollment prior to initial opioid) and we tested the model’s ability when predicting opioid use disorder from 3 months until 1 year after the initial opioid dispensing. In addition, we validated the model when only including patients who are in the database for 1 year or more post index to see whether we introduced a bias by including opioid use disorder patients who are not observed for the complete 1 year.

## Results & discussion

### Sample size

The number of eligible people in each database with an opioid for the first time with 3 years or more observation prior to the opioid was: 6,200,584, 3,955,161, 869,383 and 820,750 for CCAE, Optum, MDCD and MDCR respectively. As the target populations were very large, we randomly sampled 500,000 patients from each database as a tradeoff between having sufficient data to train the models and making model training efficient. We then excluded patients who dropped out from the database within the 1-year follow-up if they did not have the outcome recorded between 1 day after index and leaving the database (as we do not know whether they would have been recorded as having opioid use disorder if they completed follow-up). This excluded 128,296, 128,742, 156,448 and 115,576 patients from the 500,000 sample in Optum, CCAE, MDCD and MDCR respectively. If a patient had opioid use disorder recorded 1 day after index and up to leaving the database, they were not excluded.

The external validation (a model trained on one dataset but tested on a different dataset) of any model used all the data available as model application is quick. See S2 Appendix B for the development and validation attrition table.

### Data characteristics

The percentage of new users of opioids who have reported opioid use disorder within a year was 0.04% in MDCR, 0.09% in CCAE, 0.14% in Optum and 0.26% in MDCD. The percentage of opioid use disorder within a year of a first-time opioid dispensing appears to be increasing over 2002 to 2017, see S3 Appendix C. The similarity between the datasets, stratified by outcome, can be seen in Table 2.

**Table 2:** Characteristics of the four claims datasets.

	Optum		CCAE		MDCD		MDCR	
	Use Disorder:		Use Disorder:		Use Disorder:		Use Disorder:	
	No	Yes	No	Yes	No	Yes	No	Yes
Characteristic	% (n = 371,199)	% (n = 505)	% (n = 370,917)	% (n = 341)	% (n = 342,643)	% (n = 909)	% (n = 384,280)	% (n = 144)
Mean Age (sd)	48 (21)	49 (23)	38 (17)	32 (16)	27 (24)	43 (22)	76 (7)	74 (7)
Gender: female (%)	53	43	52	43	56	58	52	56
Medical history: General (%)								
Acute respiratory disease	52	49	60	65	71	65	42	51
Attention deficit hyperactivity disorder	3	8	4	13	14	14	0	0
Chronic liver disease	2	5	2	2	2	7	2	6
Depressive disorder	12	27	10	29	18	49	7	25
Human immunodeficiency virus infection	0	1	0	1	1	2	0	1
Obesity	9	10	6	7	15	21	4	13
Osteoarthritis	20	30	11	17	12	38	40	66
Renal impairment	5	15	1	3	6	15	10	21
Schizophrenia	0	1	0	0	3	6	0	1
Viral hepatitis C	0	4	0	1	1	6	0	1
Medical history: Cardiovascular disease								
Heart disease	23	28	14	13	19	36	58	64
Medical history: Neoplasms								
Malignant neoplastic disease	11	11	7	9	5	11	33	30
Medication use								
Antidepressants	18	31	18	37	16	26	22	38
Antiepileptics	7	20	6	21	12	22	12	25
Antiinflammatory and antirheumatic products	38	41	43	42	45	48	46	55
Antineoplastic agents	5	7	5	9	4	4	7	8
Drugs used in diabetes	9	10	6	7	6	4	20	24
Psycholeptics	26	36	27	35	36	53	39	56
Psychostimulants, agents used for adhd and nootropics	4	9	6	17	13	15	2	4

The results show that the percentage of patients with reported opioid use disorder within a year of first opioid prescription in the USA ranged between 0.04% to 0.26%. Those with opioid use disorder are more likely to have a history of mental health disorders such as depressive disorder, obesity, renal impairments and osteoarthritis.

### Full models

The models obtained internal validations above 0.76 using ~100–200 predictors from amongst ~80,000 covariates available, see Table 3.

**Table 3:** The data and internal model performance details.

Dataset	N	Outcome (%)	# Covariates		AUC	
			Total	Inc Model	Train (75%)	Test (25%)
**OPTUM**	371,704	505 (0.14)	82,520	203	0.85	0.77 (0.73–0.82)
**CCAE**	371,258	341 (0.09)	78,227	121	0.87	0.79 (0.74–0.84)
**MDCD**	343,552	909 (0.26)	79,025	258	0.89	0.85 (0.83–0.88)
**MDCR**	384,424	144 (0.04)	78,372	108	0.90	0.76 (0.67–0.84)

The internal validation performance (25% data test set) and external validation performances are presented as a grid in Table 4. The results show that models developed using demographics, visit types, medical conditions, prescribed drugs and procedures prior to the opioid were able to discriminate those who developed from those who didn’t develop the disorder.

**Table 4:** The AUC values for each validation. The brackets represent the confidence intervals—this is presented when the validation data is <100,000 people and ignored for larger data where the AUC values are more stable.

Development Data	Validation Data			
	OPTUM	CCAE	MDCD	MDCR
**OPTUM**	0.77 (0.73–0.82)	0.77	0.81	0.73
**CCAE**	0.72	0.79 (0.74–0.84)	0.81	0.73
**MDCD**	0.68	0.7	0.85 (0.83–0.88)	0.76
**MDCR**	0.64	0.59	0.74	0.76 (0.67–0.84)

The external validation shows that the models were generally able to transport across diverse datasets, except the model developed on MDCR, which performed poorly when applied to CCAE or Optum.

The code required to apply the model to any OMOP CDM database is available at https://github.com/OHDSI/StudyProtocols/tree/master/OpioidModels and a website enabling users to interactively view the models and performance is available at http://data.ohdsi.org/opioidExplorer/. The full models are also available to view in S1–S5 Files.

### CROUD

Consistent with previous studies, the identified predictors in the full models were substance abuse, different forms of pain (low back pain and painful neuropathy), anxiety, mood disorders/depression and age. Other predictors identified in the full models were whether the person had an ER visit near to the opioid prescription and renal impairment. A clinician and data scientist subjectively chose 8 variables based on whether the variable was selected across the different database full models and whether the variable was sufficiently common. This led to the development of CROUD, a simple score model, that asks 8 yes or no questions and assigns points based on the responses as presented in Table 5. A person's opioid use disorder risk score is calculated by summing up their points for all eight questions. The performance of CROUD was evaluated using all data available, obtaining AUCs between 0.72–0.83, see Table 7. Full cutoff performance details for CROUD are available in S4 Appendix D. The code sets used to define each CROUD variable using the OMOP CDM is available in S6 File.

**Table 5:** The CROUD model questions and points per question response. A patient’s risk can be determined by calculating their overall score as the sum of their points and then using Table 6 to match the risk for that score.

Model Question	Points if answer Yes	Points if answer No	Your Points:
**Are you age 15–29?**	+5	+0	
**Have you been to the ER within the past 30 days?**	+2	+0	
**Medical history: have you ever been diagnosed with:**			
**Substance abuse (including smoking, alcohol and recreational drugs)?**	+12	+0	
**Anxiety Disorder?**	+4	+0	
**Mood Disorder?**	+6	+0	
**Low Back Pain?**	+5	+0	
**Renal Impairment?**	+6	+0	
**Painful Neuropathy?**	+3	+0	
	**Total (sum up all your points):**		

**Table 6:** Predicted risk in each score strata compared to population risk of 0.14% in Optum.

	Observed risk of opioid use disorder (% or people with this score who will develop opioid use disorder within 1-year)	% of population in strata
**All population:**	0.15	
**Risk Score Strata:**		
**0–2**	0.06	39.7
**3–6**	0.10	26.1
**7–10**	0.16	14.9
**11–14**	0.23	9.5
**15–18**	0.35	5.3
**19–22**	0.58	2.8
**23+**	1.14	1.6

**Table 7:** External validation discriminative performance of CROUD.

Database	Number of Patients	Patients with opioid use disorder (%)	CROUD model AUC
**OPTUM (Development Data)**	2,897,134	4,262 (0.15%)	0.72
**CCAE**	4,540,979	4,376 (0.10%)	0.77
**MDCD**	579,563	1,691 (0.29%)	0.83
**MDCR**	630,022	264 (0.04%)	0.75

We performed various sensitivity analyses to determine the performance of CROUD for various settings, see S5 Appendix E. We developed CROUD using a target population of any new users of opioids as it is not always clear at the point in time when a patient is first dispensed an opioid whether they will be a long-term user or not. However, when applied to patients who are on opioids for a minimum of 90 days the AUC was 0.68, 0.65, 0.61 and 0.74 across Optum, CCAE, MDCD and MDCR respectively, see S5 Appendix E for full details. We also investigated the impact of requiring 3 years of prior observation by applying CROUD to new users of opioids with only 1-year prior observation. The AUC performance was consistent across the databases ranging between 0.73–0.75. Finally, we evaluated CROUD when predicting opioid use disorder 90 days after the first dispensing up to a year later. Although the opioid use disorder cases dropped the performance remained similar with AUCs of 0.71, 0.77, 0.82 and 0.74 for Optum, CCAE, MDCD and MDCR respectively. This suggests the model is robust across settings.

The risk score can be converted to a risk percentage using Table 6. For example, if somebody answers yes to a history of anxiety disorder (+4 points) and renal impairment (+6 points), but none of the other model variables (+0 points), then their risk score would be 10 points. The risk percentage of future opioid use disorder for somebody with a risk score of 7–10 is 0.16% which is similar to the population average. If somebody answered yes to substance abuse (being a current smoker) (+12 points), yes to renal impairment (+6 points) and yes to mood disorder (+6 points) and no to other questions (+0 points) their risk score is 24 and Table 6 shows their risk of developing opioid use disorder is 1.14% which means they are 8 times more likely to develop opioid use disorder within a year than the average person.

CROUD can be used by clinicians to objectively stratify patients into lower and higher risk groups. Clinicians may currently apply subjective risk assessments but CROUD gives them the option of using an objective assessment that has been validated across four US claims databases. In addition, CROUD may include variables that clinicians would not have considered. The current approach is to monitor every patient closely, but with CROUD the healthcare providers can objectively identify higher-risk patients and dedicate more time to the patients with higher risks of developing opioid use disorder. Overall, the risk of developing future opioid use disorder was 0.15%. However, we found that ~40% of patients were assigned a risk score of 2 or less. This group was the low risk group with only a 0.06% risk of future opioid use disorder. Patients with a risk score of 11 or higher were the moderate risk group, making up ~20% of the opioid new users, but having a future opioid use disorder risk of >0.2% (greater than the population average). Only 1.6% of patients had a risk score of 23 or more; collectively, these patients had a 1.14% risk of future opioid use disorder, 8 times the population average.

Identifying patients with a CROUD score of 11 or greater would lead to a sensitivity of 51%, specificity of 81% and positive predictive value (PPV) of 0.4%, whereas identifying patients with a CROUD score of 23 or greater would lead to a sensitivity of 13%, specificity of 98% and PPV of 1.1%. As only 0.15% of the population have opioid use disorder, the patients with a CROUD score of 11 or greater have 3 times the risk of the average patient and those with a CROUD score of 23 or greater have 8 times the risk of the average patient. The performances at different score cut offs can be found in S4 Appendix D.

CROUD could be used by clinicians at the point of first opioid dispensing to determine a patient’s personalized risk. The advantage of developing a model that can be applied at the point in time before the first opioid is that it enables a preventative intervention to be implemented as the people are free of the disorder, whereas models that are implemented after the first opioid may identify when a patient is already displaying behaviors of opioid use disorder and the use disorder can only be treated and not prevented. The Center for Disease Control and Prevention (CDC) guidelines now recommends that periodic urinary testing (testing at the start and annually for chronic users) is implemented [19]. CROUD can personalize the risk of developing use disorder and therefore the model could be used to personalize urine testing. For example, low risk patients (those with a score or 2 or less) could have fewer tests which would save clinicians time. In addition, the Food and Drug Administration (FDA) has requested manufacturers of opioids to provide continuing education to address the misuse and abuse of opioids. In the FDA blueprint it is recommended that the overall opioid treatment approach and plan would be well documented in written agreements for every patient [20]. CROUD can be applied the first time a patient is about to be prescribed an opioid to objectively risk stratify the patient, and this could be used to personalize the treatment plan. Patients at higher risk could be dedicated more time/resources. This may help clinicians who often have to make subjective assessments. Although the model discrimination was good, due to the low incidence of recorded opioid use disorder, there is a high false positive rate. This means the majority of the patients identified as high risk will not have opioid use disorder and therefore the model should not be used as contraindication or to restrict opioid utilization.

CROUD is simple to implement and can be use in opioid naïve populations. We recommend using CROUD before a patient is first prescribed an opioid. If a clinician wishes to continue using risk models to objectively assess risk after 1 year, we suggest using existing published opioid use disorder models [6,9] to continuously monitor a patient who is a long-term user of opioids.

The main limitation of this study is that we used the recording of opioid abuse/dependency as a proxy for opioid use disorder, so what we capture may fall into the severe end of the opioid use disorder spectrum. This may also lead to incorrect labels (misclassification). In addition, opioid use disorder may not be noticed or recorded by medical professionals, so the incidence reported in this paper may be an underestimate. Death due to opioid use disorder may not get recorded as opioid use disorder, so these cases may not get captured in the data. Although opioid use disorder may be under-reported, as long as those with the disorder who get recorded are not systematically different from those with the disorder unrecorded, then the model should still be applicable. It is not possible to test whether those with opioid use disorder unrecorded are different from those with it recorded. In addition, the under-reporting of opioid use disorder would be a limitation of any prediction model, but a model that can identify those who have recorded opioid use disorder (potentially due to being severe or seeking help for use disorder) is still useful. Another limitation when applying the model to claims data is that clinically relevant information is often under recorded in the data. For example, lifestyle variables such as smoking and alcohol intake are rarely recorded in claims, but these appear to be informative in predicting opioid use disorder. In this paper we validated CROUD on claims data but did not investigate self-reported data. Clinicians should use a patient’s medical records for an accurate risk score when implementing CROUD. In future work it would be interesting to see whether CROUD generalizes to self-reported data. Finally, the rate of recorded opioid abuse in the databases appears to be increasing over time. In this paper we have not explored temporal changes; this is an interesting area of future work as temporal changes can limit any prediction model.

## Conclusion

In this paper CROUD, an 8-question score-based model, was developed to predict the risk of opioid use disorder at the point of first opioid dispensing. CROUD only used age, presence of low back pain, substance abuse, mood disorder, anxiety disorder, renal impairment, painful neuropathy and recent ER visit to obtain AUCs between 0.72–0.82 in predicting future opioid use disorder. CROUD could be used by clinicians to assess a patient’s personalized risk of opioid use disorder as it just requires knowledge of their medical history. In addition, CROUD appears to be generalizable to a wide population and shows promise at being able to help make clinicians more informed and enable them to objectively target or personalize new opioid dispensing guidelines.

## Supporting information

S1 File (CSV)

S2 File (CSV)

S3 File (CSV)

S4 File (CSV)

S5 File (CSV)

S6 File (CSV)

S1 Appendix Cohort definitions. (DOCX)

S2 Appendix Attrition table. (DOCX)

S3 Appendix Incidence of opioid use disorder recorded within a year of the initial opioid by year of opioid dispensing across the datasets. (DOCX)

S4 Appendix Cut-off performance of CROUD on the Optum dataset. (DOCX)

S5 Appendix CROUD sensitivity performance. (DOCX)

S6 Appendix Expanded methodology. (DOCX)
