# Supplementary material for: Wisdom of the CROUD: Development and validation of a patient-level prediction model for opioid use disorder using population-level claims data
Source: PLoS One. 2020 Feb 13;15(2):e0228632. doi: 10.1371/journal.pone.0228632 (PMC7017997; doi:10.1371/journal.pone.0228632)
Supplement: S1 Appendix — (DOCX) [file pone.0228632.s007.docx]

Appendix

***A: Cohort definitions***

Target Population:

Primary criterion:

A drug era of opioid (RxNorm: 5489 Hydrocodone, ATC: N02A OPIOIDS and N02AA Natural opium alkaloids plus all descendants) for the first time in the person's history

Additional Inclusions:

- continuous observation of at least 1095 days prior to index date
- No Opioid abuse any time prior (snomed: 5602001 Opioid abuse plus descendants)
- No Opioid dependence any time prior (snomed: 75544000 Opioid dependence plus descendants)
- No opioid withdrawal drug any time prior (RxNorm: 352291 Buprenorphine 8 MG / Naloxone 2 MG Sublingual Tablet [Suboxone], 352990 Suboxone, 575988 Buprenorphine 2 MG / Naloxone 0.5 MG [Suboxone], 575989 Buprenorphine 8 MG / Naloxone 2 MG [Suboxone], 352290 Buprenorphine 2 MG / Naloxone 0.5 MG Sublingual Tablet [Suboxone], 1180238 Suboxone Oral Product, 1180239 Suboxone Pill, 1297024 Suboxone Sublingual Product, 1296320 Suboxone Oral Strip Product, 370347 Buprenorphine / Naloxone Sublingual Tablet [Suboxone] plus descendants)
- No methadone pain clinics any time prior (snomed: 310653000 Drug addiction therapy – methadone, 200021000000100 Drug add detox ther methadone; ICD10PCS: HZ81ZZZ Medication Management for Substance Abuse Treatment, Methadone Maintenance, HZ91ZZZ Pharmacotherapy for Substance Abuse Treatment, Methadone Maintenance, HZ91Z Substance Abuse Treatment @ None @ Pharmacotherapy @ Methadone Maintenance @ None, HZ81ZZ Substance Abuse Treatment @ None @ Medication Management @ Methadone Maintenance @ None @ None, HZ91 Substance Abuse Treatment @ None @ Pharmacotherapy @ Methadone Maintenance, HZ81 Substance Abuse Treatment @ None @ Medication Management @ Methadone Maintenance, HZ81Z Substance Abuse Treatment @ None @ Medication Management @ Methadone Maintenance @ None, HZ91ZZ Substance Abuse Treatment @ None @ Pharmacotherapy @ Methadone Maintenance @ None @ None plus descendant)
- No opioid or potential opioid drug abuse any time prior (single snomed codes: 191821007 Opioid dependence in remission, 191936009 Nondependent mixed drug abuse, continuous, 26416006 Drug abuse, 75544000 Opioid dependence, 191934007 Nondependent mixed drug abuse, 191816009 Drug dependence, 191820008 Episodic opioid dependence, 191937000 Nondependent mixed drug abuse, episodic; snomed codes: 191819002 Continuous opioid dependence and 191865004 Combined opioid with other drug dependence plus descendants)

Opioid use disorder:

- a condition occurrence of [RWE CTF] Opioid abuse (snomed: 5602001 Opioid abuse plus descendants and snomed: 75544000 Opioid dependence plus descendants)
- a drug exposure of opioid withdrawal drug (RxNorm: 352291 Buprenorphine 8 MG / Naloxone 2 MG Sublingual Tablet [Suboxone], 352990 Suboxone, 575988 Buprenorphine 2 MG / Naloxone 0.5 MG [Suboxone], 575989 Buprenorphine 8 MG / Naloxone 2 MG [Suboxone], 352290 Buprenorphine 2 MG / Naloxone 0.5 MG Sublingual Tablet [Suboxone], 1180238 Suboxone Oral Product, 1180239 Suboxone Pill, 1297024 Suboxone Sublingual Product, 1296320 Suboxone Oral Strip Product, 370347 Buprenorphine / Naloxone Sublingual Tablet [Suboxone] plus descendants)
- a procedure of methadone pain clinics (snomed: 310653000 Drug addiction therapy – methadone, 200021000000100 Drug add detox ther methadone; ICD10PCS: HZ81ZZZ Medication Management for Substance Abuse Treatment, Methadone Maintenance, HZ91ZZZ Pharmacotherapy for Substance Abuse Treatment, Methadone Maintenance, HZ91Z Substance Abuse Treatment @ None @ Pharmacotherapy @ Methadone Maintenance @ None, HZ81ZZ Substance Abuse Treatment @ None @ Medication Management @ Methadone Maintenance @ None @ None, HZ91 Substance Abuse Treatment @ None @ Pharmacotherapy @ Methadone Maintenance, HZ81 Substance Abuse Treatment @ None @ Medication Management @ Methadone Maintenance, HZ81Z Substance Abuse Treatment @ None @ Medication Management @ Methadone Maintenance @ None, HZ91ZZ Substance Abuse Treatment @ None @ Pharmacotherapy @ Methadone Maintenance @ None @ None plus descendant)
