# Supplementary material for: Wisdom of the CROUD: Development and validation of a patient-level prediction model for opioid use disorder using population-level claims data
Source: PLoS One. 2020 Feb 13;15(2):e0228632. doi: 10.1371/journal.pone.0228632 (PMC7017997; doi:10.1371/journal.pone.0228632)
Supplement: S2 Appendix — (DOCX) [file pone.0228632.s008.docx]

Appendix B: Attrition table

Table 7 The number of patients remaining at each criteria stage (included) and the number of patients excluded at each criteria stage (excluded). T corresponds to the total population and O is the number of patients with the outcome.

| Development | | | | | | | | |
| --- | --- | --- | --- | --- | --- | --- | --- | --- |
|  | CCAE | | Optum | | MDCD | | MDCR | |
| Inclusion Criterion | Included | Excluded | Included | Excluded | Included | Excluded | Included | Excluded |
| Opioid for the first time with 3 years or more observation | T: 6,200,584 O: 4,376 |  | T: 3,955,161 O: 4,262 |  | T: 869,383 O: 1,691 |  | T: 820,750 O: 264 |  |
| Sample | T: 500,000 O: 505 | T: 5,700,584 O: 3,871 | T: 500,000 O: 341 | T: 3,455,161 O: 3921 | T: 500,000 O: 909 | T: 3,693,83O: 782 | T: 500,000 O: 144 | T: 3,207,50O: 120 |
| Had opioid use disorder within 1-year post index **or** observed for 1-year post index | T: 371,704 O: 505 | T: 128,296 O: 0 | T: 371,258 O: 341 | T: 128,742 O: 0 | T: 343,552 O: 909 | T: 156,448 O: 0 | T: 384,424 O: 144 | T: 115,576 O: 0 |
| Validation | | | | | | | | |
| Opioid for the first time with 3 years or more observation | T: 6,200,584 O: 4,376 |  | T: 3,955,161 O: 4,262 |  | T: 869,383 O: 1,691 |  | T: 820,750 O: 264 |  |
| Had opioid use disorder within 1-year post index **or** observed for 1-year post index | T: 4,540,979O: 4,376 | T: 1,659,605 O: 0 | T: 2,897,134 O: 4,262 | T: 1,058,027O: 0 | T: 579,563 O: 1,691 | T: 289,820O: 0 | T: 630,022 O: 264 | T: 190,728 O: 0 |
