# Supplementary material for: Wisdom of the CROUD: Development and validation of a patient-level prediction model for opioid use disorder using population-level claims data
Source: PLoS One. 2020 Feb 13;15(2):e0228632. doi: 10.1371/journal.pone.0228632 (PMC7017997; doi:10.1371/journal.pone.0228632)
Supplement: S3 Appendix — (DOCX) [file pone.0228632.s009.docx]

***Appendix C. Incidence of Opioid use disorder recorded within a year of the initial opioid by year of opioid dispensing across the datasets.***


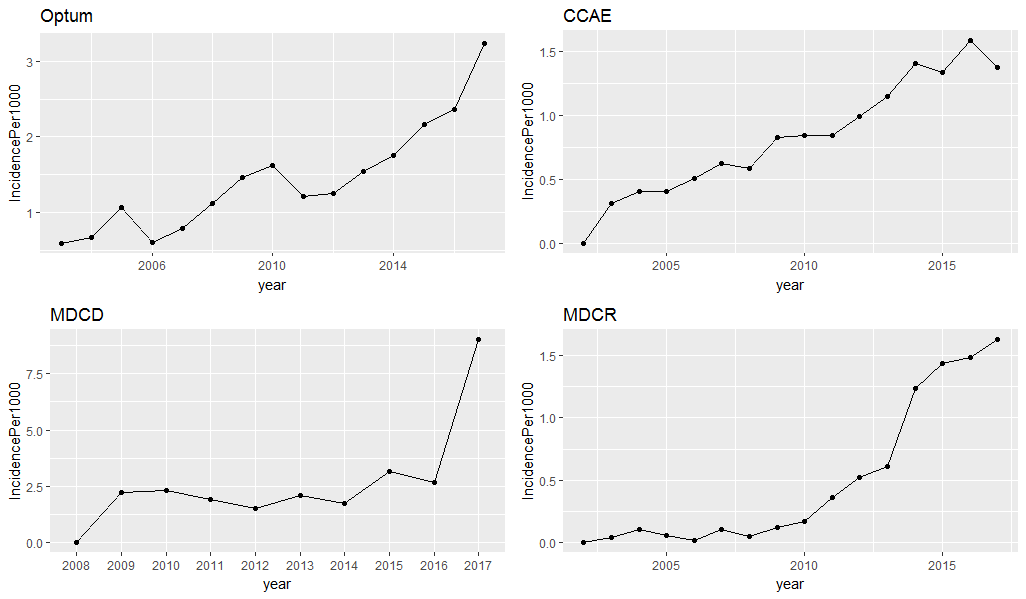


Figure 2: Incidence of Opioid use disorder within 1 year of initial opioid dispensing across time (x is year of opioid dispensing, y is incidence per 1000 patients)
