# Supplementary material for: Wisdom of the CROUD: Development and validation of a patient-level prediction model for opioid use disorder using population-level claims data
Source: PLoS One. 2020 Feb 13;15(2):e0228632. doi: 10.1371/journal.pone.0228632 (PMC7017997; doi:10.1371/journal.pone.0228632)
Supplement: S4 Appendix — (DOCX) [file pone.0228632.s010.docx]

***Appendix D. Cut-off performance of CROUD on the Optum dataset***

**The performance at various thresholds for CROUD, see Figure 1 to see the performance for every possible threshold.**

| Score Threshold | Sensitivity | Specificity | PPV | NPV |
| --- | --- | --- | --- | --- |
| 3+ | 84.7 | 39.7 | 0.21 | 99.9 |
| 7+ | 67.6 | 65.8 | 0.29 | 99.9 |
| 11+ | 51.2 | 80.8 | 0.39 | 99.9 |
| 15+ | 36.4 | 90.2 | 0.55 | 99.9 |
| 19+ | 23.7 | 95.6 | 0.78 | 99.9 |
| 23+ | 12.6 | 98.4 | 1.14 | 99.9 |


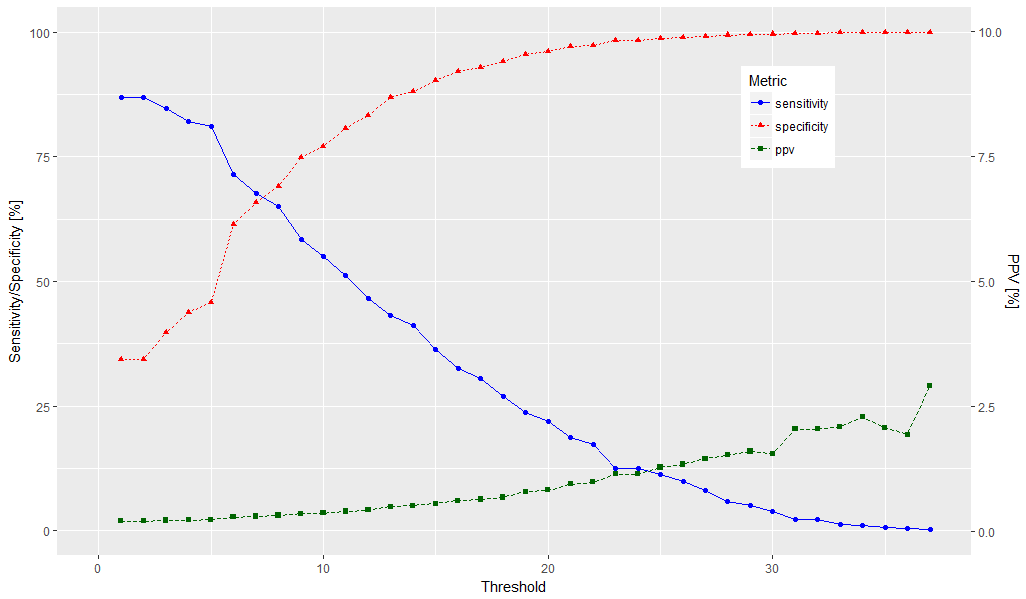


Figure 1: Threshold performance plot for CROUD
