# Supplementary material for: Wisdom of the CROUD: Development and validation of a patient-level prediction model for opioid use disorder using population-level claims data
Source: PLoS One. 2020 Feb 13;15(2):e0228632. doi: 10.1371/journal.pone.0228632 (PMC7017997; doi:10.1371/journal.pone.0228632)
Supplement: S5 Appendix — (DOCX) [file pone.0228632.s011.docx]

***Appendix E. CROUD sensitivity performance***

Applying the CROUD model to different target populations:

| Target Population | Optum | CCAE | MDCD | MDCR |
| --- | --- | --- | --- | --- |
| Patients given opioid for first time with no history of opioid use disorder and a minimum of 365 days prior observation | T: 7,670,830  O: 13,919 (0.18%)  AUC: 0.73 | T: 12,953,284  O: 17,587 (0.14%)  AUC: 0.75 | T: 1,872,669  O: 13,806 (0.74%)  AUC: 0.75 | T: 1,725,052  O: 894 (0.05%)  AUC: 0.74 (0.72-0.77) |
| Patients given opioid for first time for a least 90 days with no history of opioid use disorder and a minimum of 1095 days prior observation | T: 48, 780  O: 657 (1.3%)  AUC: 0.68 (0.66-0.70) | T: 23,677  O: 338 (1.4%)  AUC: 0.65 (0.62-0.68) | T: 5,130  O: 90 (1.8%)  AUC: 0.61 (0.56-0.67) | T: 15,662  O: 31 (0.2%)  AUC: 0.74 (0.66-0.82) |
| Patients given opioid for first time with no history of opioid use disorder or opioid abuse recorded within 90 days after and a minimum of 1095 days prior observation | T: 2,895,144  O: 3,073 (0.1%)  AUC: 0.71 | T: 4,538,971  O: 3,293 (0.07%)  AUC: 0.77 | T: 578,536  O: 1,174 (0.2%)  AUC: 0.82 | T: 629,867  O: 151 (0.02%)  AUC: 0.74 |
| Patients given opioids for the first time with no history of opioid use disorder and a minimum of 3 years prior observation and 1-year post observation (patients who died within the year follow-up excluded) | T: 2,896,369  O: 3,496  AUC: 0.72 | T: 4,540,179  O: 3,574  AUC: 0.77 | T: 579,111  O: 1,238  AUC: 0.82 | T: 629,956  O: 198  AUC: 0.76 |
