# Supplementary material for: Wisdom of the CROUD: Development and validation of a patient-level prediction model for opioid use disorder using population-level claims data
Source: PLoS One. 2020 Feb 13;15(2):e0228632. doi: 10.1371/journal.pone.0228632 (PMC7017997; doi:10.1371/journal.pone.0228632)
Supplement: S6 Appendix — (DOCX) [file pone.0228632.s012.docx]

***Appendix F: Expanded methodology***

Extracting the data

To train the full models we first create the labelled dataset. For each patient dispensed an opioid with no prior recorded opioid use disorder, we construct variables based on his medical records prior to, or on the day of, the opioid dispensing. For examples, if a patient has type 2 diabetes recorded in the prior 60 days, then he will have a value of 1 for the variable “type 2 diabetes in prior 365 days”, if the patient has no record of fluoxetine prior to the opioid dispensing, then he will have a value of 0 for the variable “drug fluoxetine in prior 365 days” and a value of 0 for “drug fluoxetine any time prior”. We construct similar variables for any condition, drug, observation, measurement or procedure recorded for any patient dispensed an opioid with no prior recorded opioid use disorder. We then determine the labels by seeing whether each patient has opioid use disorder recorded within a year of the initial opioid dispensing. For example, if a patient has use disorder recorded 148 days after the initial opioid, then he will be labelled as 1. If a patient has no use disorder recorded within the year after the initial opioid, then he will be labelled 0.

Sampling the data

As the data available to train a model increases in size, the model performance increases but at a certain point the performance converges to a maximum and adding more data does not increase performance. Instead, the additional data just slows down model training. For efficiency, we sampled 500,000 patients as this will likely result in a model that is optimal (or close) but will make model training more efficient. We randomly sampled by generating a number uniformly between 0 and 1 for each patient and picking the patients with the largest 500,000 numbers

Training the full models

Using the 500,000 sampled labeled data we lost some of these patients due to them leaving the database in the 1-year follow-up. The remaining labelled data is then split into train (data used to develop the model) and test (data not used in model development and only used to evaluate the model) sets. We decided to use 75% of the data in the train set and 25% in the test set. There are two different things that need to be optimized when training a lasso logistic regression, the variance hyper-parameter (regularization – the cost of model complexity) and the model parameters beta coefficients. We pick the optimal hyper-parameter by implementing 3-fold cross validation on all the training data, this works as follows: first we split the training data into 3 disjoint sets, then we want to assess the performance of models for different hyper-parameters by training models for each hyper-parameter setting 3 times (each time using 2 different combinations of the 3 disjoin sets) and evaluating on the left out disjoint set, then we combine the 3 left out disjoint sets predictions to calculate the overall performance of the model for the specific hyper-parameter setting. We used software that does an automatic search for the variance hyper-parameter by focusing on values near to where performance has done well and then picked the variance hyper-parameter value that lead to the best performance. Finally, we find the optimal beta coefficients for the lasso logistic regression by training on the full training data with the previously identified hyper-parameter value set.

Evaluating the models

We then implement the trained model on the test set and compare the predicted risk with the ground truth of whether each patient had the outcome or not. The overall measure of discrimination is measured by the area under the receiver operating characteristic curve (AUC) and corresponds to the probability that you assign a higher risk to a randomly selected patient who has the outcome compared to the risk assigned to a randomly selected patient that doesn’t. We can also evaluate the model’s performance when classifying patients into those who will have the outcome and those who will not, this is threshold dependent and can be modified depending on the desired operating characteristics.

Developing CROUD

We provide links and instructions on how to implement the full models, but these are likely to be too complex for a clinical setting. Therefore, we decided to create a simple model that uses a reduced number of variables. To accomplish this, we found variables that were consistently selected into the full models across datasets using the 500,000-patient sample (i.e., the variable was included in the models developed on all four databases). We then excluded variables that were rare and combined similar variables (e.g., smoking, alcohol and substance abuse were combined into one variable). This resulted in the 8 variables used by CROUD. To determine the points for each variable, we trained a logistic regression model that only included the 8 variables using the same 500,000 patient sample used by the full model in Optum. We then rounded and scaled the coefficients to obtain simple integer points. For example, if the logistic regression coefficients were 0.3, 1.3, 0.7, 0.05, 0.1, 0.1, 1.1 and 0.4 then multiplying by 100 would give: 30, 130, 70, 5, 10, 10, 110 and 40, we could then divide by 5 to get: 6, 26, 14, 1, 2, 2, 22 and 8 which is the smallest scaled integer values. As we only used a 500,000-patient sample to develop the full and simple models, we were then able to fairly evaluate the CROUD 8-variable model on the full data to obtain the discriminative performance and ensure we didn’t overfit.

Calculating the risk for CROUD scores

To calculate the risks for each score we simply calculated how many patients in the full Optum data had the score and also had opioid use disorder recorded within a year and then divided by the number of patients in the full Optum data who had the score. Similar scores often had similar risk, so we combined these into one group where possible. For example, if a score of 1 had a risk of 0.0098 and a score of 2 had a risk of 0.012, then we combined these together, so a score of 1-2 had a risk of 0.01.
